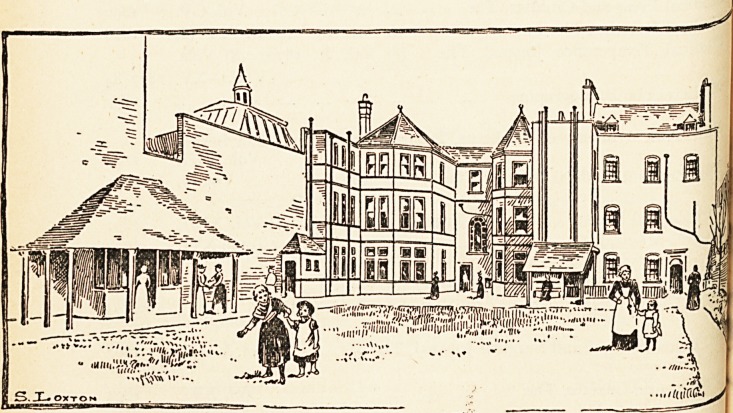# The Bristol Eye Hospital

**Published:** 1893-06

**Authors:** 


### The Bristol Eye Hospital.

This Institution, which was founded in the early part of the century, has in the past few years undergone very considerable enlargement, and by the recent addition of a new wing has been made a very complete and convenient Hospital for the study and treatment of diseases of the eye.

The frontage in Lower Maudlin Street indicates that the building consisted of two adjoining houses: one of these constituted the Hospital from 1810 until 1886, when the second house was added; more recently the two have been modified and enlarged by throwing out bows towards the gardens at the back, as shown in the illustration, for which we are indebted to the courtesy of the Editor of the *Western Daily Press.*

The ground floor is occupied by the out-patients' department, which consists of a large entrance hall, a waiting-room convenient for two hundred people, a dispensary, and the consulting and ophthalmoscope rooms of the Surgeon and Assistant-Surgeons. The first floor, reached by a fine old carved staircase, comprises the board-room and museum, the operating-room, apartments for the Matron and for the Resident House Surgeon, and two sitting-rooms—one for the male and one for the female patients. Above are three large wards, each extending the whole depth of the Hospital, and looking out back and front respectively upon the hospital garden and that of the Royal Infirmary. The wards contain twenty-six beds; in close proximity are bath-rooms for the patients and a nurses' room.

The Hospital has two staircases: one side of the building is occupied by the wards and sitting-room of the male patients; the other part is used entirely by the women and children.

All the closets have been placed in stacks built out from the general structure, and the drainage is thoroughly isolated.

More than 38,000 patients have been under treatment during the past ten years. The average of 4,000 a year has been lately exceeded, and there have been from 16,000 to 17,000 separate visits.

## Figures and Tables

**Figure f1:**